# Molecular-Morphological Relationships of the Scaffold Protein FKBP51 and Inflammatory Processes in Knee Osteoarthritis

**DOI:** 10.3390/cells10092196

**Published:** 2021-08-25

**Authors:** Fabián Poletti, Rebeca González-Fernández, María-del-Pino García, Deborah Rotoli, Julio Ávila, Ali Mobasheri, Pablo Martín-Vasallo

**Affiliations:** 1 Laboratorio de Biología del Desarrollo, UD de Bioquímica y Biología Molecular Instituto de Tecnologías Biomédicas de Canarias, Universidad de La Laguna, La Laguna, Av. Astrofísico Sánchez s/n, 38206 La Laguna Tenerife, Spain; fabypoletti@hotmail.com (F.P.); refernan@ull.edu.es (R.G.-F.); deborah_rotoli@yahoo.it (D.R.); javila@ull.edu.es (J.Á.); 2 Orthopaedic Surgery and Trauma Unit, Royal Berkshire Hospital NHS Foundation Trust, Reading RG1 5AN, UK; 3 Unidad de Cirugía Ortopédica y Traumatología, Hospital San Juan de Dios-Tenerife, Ctra. Santa Cruz Laguna 53, 38009 Santa Cruz de Tenerife, Spain; 4 Department of Pathology, Eurofins® Megalab-Hospiten Hospitals, 38001 Santa Cruz de Tenerife, Spain; mpgarcias@megalab.es; 5 Institute of Endocrinology and Experimental Oncology (IEOS), CNR-National Research Council, 80131 Naples, Italy; 6 Research Unit of Medical Imaging, Physics and Technology, Faculty of Medicine, University of Oulu, 90570 Oulu, Finland; ali.mobasheri@oulu.fi; 7 Department of Regenerative Medicine, State Research Institute Centre for Innovative Medicine, LT-08406 Vilnius, Lithuania; 8 Departments of Orthopedics, Rheumatology and Clinical Immunology, University Medical Center Utrecht, 3584 CX Utrecht, The Netherlands; 9 Department of Joint Surgery, The First Affiliated Hospital of Sun Yat-Sen University, Guangzhou 510080, China; 10 World Health Organization Collaborating Center for Public Health Aspects of Musculoskeletal Health and Aging, Université de Liège, B-4000 Liège, Belgium

**Keywords:** osteoarthritis, knee, FKBP51, scaffold proteins, chondrocytes, synovial membrane, Hoffa’s fat pad, low-grade inflammation, nerve fiber

## Abstract

Knee osteoarthritis (OA) is one of the most prevalent chronic conditions affecting the adult population. OA is no longer thought to come from a purely biomechanical origin but rather one that has been increasingly recognized to include a persistent low-grade inflammatory component. Intra-articular corticosteroid injections (IACSI) have become a widely used method for treating pain in patients with OA as an effective symptomatic treatment. However, as the disease progresses, IACSI become ineffective. FKBP51 is a regulatory protein of the glucocorticoid receptor function and have been shown to be dysregulated in several pathological scenario’s including chronic inflammation. Despite of these facts, to our knowledge, there are no previous studies of the expression and possible role of FKBP51 in OA. We investigated by double and triple immunofluorescence confocal microscopy the cellular and subcellular expression of FKBP51 and its relations with inflammation factors in osteoarthritic knee joint tissues: specifically, in the tibial plateau knee cartilage, Hoffa’s fat pad and suprapatellar synovial tissue of the knee. Our results show co-expression of FKBP51 with TNF-α, IL-6, CD31 and CD34 in OA chondrocytes, synovial membrane cells and adipocytes in Hoffa’s fat pad. FKBP51 is also abundant in nerve fibers within the fat pad. Co-expression of FKBP51 protein with these markers may be indicative of its contribution to inflammatory processes and associated chronic pain in OA.

## 1. Introduction

Knee osteoarthritis (OA) is one of the most prevalent chronic conditions in the adult population worldwide, affecting over 80% of the population beyond the age of 55 [1]. According to recent studies knee OA is not only one of major causes of impairment to being able to carry out daily activities in older adults, but it is also one of the major causes of physical disability in adults, in addition to being associated with higher rates of depression and higher rates of chronic non-communicable diseases [2].

The current non-surgical and purely symptomatic treatment of OA mainly consists of activity modifications, weight reduction, physiotherapy, non-steroidal anti-inflammatory drugs, intraarticular corticosteroids, and hyaluronic acid injections, according to the latest international guidelines [3]. We are still not able to stop or significantly slow the progression of the disease [4]. End of stage OA is commonly treated with a knee replacement surgery [5].

Even though some experimental disease-modifying therapies (DMOADs), such as chondroitin sulfate and glucosamine, have shown promising results in the preclinical phase, none of these DMOADs have been able to reproduce these results in clinical trials [6]. This is thought to be due to an incomplete understanding of the underlying mechanisms that cause OA. OA is no longer considered as a purely mechanical process but rather one that has been increasingly recognized to include chronic and persistent low-grade inflammation [7]. This is thought to be the result of the imbalance between pro and anti-inflammatory mediators that induce extracellular matrix (ECM) degeneration and chondrocyte apoptosis, subchondral bone remodeling, osteophyte formation, inflammatory infiltration of the synovial tissues and the infrapatellar fat pad, as well as neo angiogenesis and nerve invasion of the articular cartilage and menisci [8,9]. In this context the release of the cytokines tumor necrosis factor α (TNF-α) and interleukin-6 (IL-6) have been shown to be particularly harmful for the joint tissue cells due to their catabolic and apoptotic effects [9]. Furthermore, both molecules are used as therapeutic targets for rheumatic and inflammatory joint diseases [10,11]. Based on these facts, we chose anti-TNF-α and anti-IL-6 antibodies as markers of inflammation. CD31, CD34, and Iba1 were selected as markers for endothelial, pluripotential, or macrophage cells in order to check angiogenesis, linking progenitor and adult stem cell phenotypes and microenvironmental cells involved in inflammation, respectively [12,13,14].

OA affects not only the cartilage that covers the surfaces of the joint but all the structures of the synovial joint, including the synovial membrane, the subchondral bone, the menisci, and Hoffa’s fat pad [15,16]. Hoffa’s fat pad fills the space between patella, femur, meniscus and tibia, in order to absorb impulsive actions generated in the joint and to distribute the synovial fluid [17]. In OA, Hoffa’s fat pad is usually affected by inflammation, hypertrophy, and fibrosis [18]. Thin nerve fibers and free nerve endings along vessels and among adipocytes have been related with proprioception, pain and with the time it takes to recover after knee arthroplasty [19]. Microtubule-associated protein 2 (MAP2) is a neuron-specific cytoskeletal protein, rich in dendrites [20]. Antibodies to MAP2 are used to identify neuronal cells and trace dendritic processes. Terminal endings in Hoffa’s fat pad have been associated either in the mechanical, inflammatory, or degenerative origin of knee pain [21].

In addition to the above, emerging research is showing that OA may share some common inflammatory signaling and molecular pathways with neoplastic disease [22]. In this context, scaffold proteins (scaffolins or scaffoldins) are crucial regulators of many key signaling pathways [23,24,25,26]. However, very little is known about the implications of scaffold proteins in the development of OA.

The scaffolding protein FK506-binding protein 51 (FKBP51) is a peptidyl-prolyl isomerase (PPI) and a multi-functional immunophilin that catalyzes the cis-trans transformation of peptidylprolyl imide bonds in target proteins [24]. There are three distinct proteins form this group: FKBP12, FKBP51, and FKBP52 [24]. FKBP51 commutes among mitochondria, cytoplasm, and nucleus, modulating several signaling pathways and is considered a molecular integrant of the adaptation process and a co-chaperone [25,26]. FKBP51 exerts relevant roles through its effects on steroid receptor maturation and through its regulation of several signaling pathways, such as NF-kB [27], PKA [28], transforming growth factor β (TGF β) and Akt [29,30,31]. Expression of the protein FKBP51 has been shown to be dysregulated in several pathological scenarios, including cancer, depression, and chronic inflammation [27,28,32,33,34,35,36,37,38]. In addition, recent preclinical and clinical studies suggest an important role for the protein FKBP51 in the generation and maintenance of many chronic pain status [39,40]. As chronic inflammation and chronic joint pain are key elements of the osteoarthritic process it would be interesting to study the role of the protein FBKP51 in the inflammatory process and the chronic pain status that accompanies the osteoarthritic process. To our knowledge, there are no previous studies on the expression of FKBP51 in the osteoarthritic joint tissues of the human knee.

In this study we hypothesized that FKBP51 is involved in the inflammatory and pain mechanisms in OA. We present novel data showing the cellular and subcellular expression of FKBP51 and its relations with inflammatory factors in osteoarthritic knee joint tissues: specifically, in the tibial plateau knee cartilage, Hoffa’s fat pad, and suprapatellar synovial tissue of the knee.

## 2. Materials and Methods

### 2.1. Patients

The knee tissue samples were collected from a population presenting with idiopathic end of stage OA, and the end of stage OA was diagnosed following the American College of Rheumatology clinical and radiological criteria. The population was recruited from the total knee replacement waiting list of San Juan de Dios Hospital, Tenerife, Spain during the first semester of the year 2019. Four males and 10 females aged between 50 and 85, with symptomatic end of stage OA were included (Table 1). Patients were excluded if they had rheumatoid arthritis, were on treatment with corticosteroids, suffered any mental health condition, had history of previous drug or alcohol abuse, had previous neurological or concomitant musculoskeletal disorders, or were unwilling to sign the informed consent.

The study was approved by the Ethics Committee of La Laguna University (ULL) and Hospital Universitario Nuestra Señora de Candelaria (Santa Cruz de Tenerife) (No. 198/2018, approved on 16 September 2018) in accordance with the World Medical Association ethical guidelines and the Declaration of Helsinki. All patients signed an informed consent for diagnosis and research on tissue specimen before taking part in the project.

### 2.2. Tissue Samples

Histological samples of tibial plateau cartilage, suprapatellar synovium and Hoffa’s fat pad were collected during standard total knee arthroplasty surgery. No variations on the standard surgical technique used in San Juan de Dios Hospital Tenerife, Spain were required in order to collect the samples. Tibial plateau osteotomy, Hoffa’s fat pad and suprapatellar synovium resection are standard steps performed during total knee replacement surgery in San Juan de Dios Hospital, Tenerife, Spain.

Synovial membrane tissue in close contact with infrapatellar fat pad was not included because resecting that synovium is not part of the surgical procedure used when performing a total knee replacement. In addition, meniscal tissue was not collected because studied patients where stage IV knee OA and meniscal tissue was not always available for collection.

### 2.3. Immunohistochemistry

Five-micron thick tissue frozen sections were removed from the freezer and immediately fixed with ice cold acetone for 10 min. Double or triple immunofluorescence simultaneous staining: After permeabilization in PBS1x containing 1% animal serum and 0.4% Triton X-100 for 30 min and block 1 h into PBS1x with 0.05 Triton X-100 and 10% animal serum, tissue sections were incubated simultaneously with a mixture of two (or three) distinct primary antibodies (e.g., rabbit against human target 1, mouse against human target 2, and goat against target 3) overnight at 4 °C. Samples incubated without primary antibodies were used as the negative control. Slides were incubated for 1 h at room temperature in dark with a mixture of two/three secondary antibodies raised in different species and conjugated to different fluorochromes (i.e., FITC-conjugated against rabbit- Sigma and DyLight^®^650-conjugated against mouse-Abcam). Slides were mounted with ProLong^®^Diamond Anti-fade Mountant with DAPI (Molecular Probes by Life technologies) to visualize cell nuclei.

### 2.4. Microscopy

Slides were analyzed using Leica SP8 (Leica Microsystems, Wetzlar, Germany) confocal microscope. Confocal microscopy was chosen in order to perform a cell-by-cell analysis of the distribution, localization, and intensity of expression at the protein level in specific cellular compartments, within cell types of a given tissue sample. The cell-by-cell study demonstrates higher reliability than other techniques that take the tissues with several kind of cells as a whole and homogeneous sample.

### 2.5. Antibodies

Rabbit polyclonal antibody against FKBP51 (dilution 1:50; #ab46002; Abcam, Cambridge, UK); mouse monoclonal anti-human cluster of differentiation (CD)31 (ready-to-use; #IR610; Dako, Glostrup, Denmark); mouse monoclonal anti-human CD34 Class II Clone QBEnd10 (ready-to-use; #IR632, Dako); goat polyclonal antibody against Iba1 (dilution 1:500; #ab107159 Abcam, Cambridge, UK); mouse monoclonal anti-Microtubule Associated Protein-2 (MAP2) (dilution 1:500; #MAB378 Chemicon International, Temecula, CA, USA); mouse monoclonal Anti-TNF-α (52B83) (dilution, 1:150; #sc- 52746 Santa Cruz Biotechnology Inc., Dallas, TX, USA); mouse monoclonal Anti- interleukin 6 (IL-6 (NYRhIL6)) (dilution, 1:200; #sc- 73319 Santa Cruz Biotechnology Inc., Dallas, TX, USA). Due to the higher possibility to find epitopes, we chose primary polyclonal antibodies when available and when their signal was clearer and background staining was lower.

### 2.6. Secondary Antibodies

For the secondary antibodies, we used fluorescein isothiocyanate (FITC)-conjugated goat polyclonal antibody against rabbit IgG (dilution 1:200; #F9887; Sigma-Aldrich); goat polyclonal antibody against mouse IgG DyLight 650 (dilution 1:100; #ab97018; Abcam); and Cy3 Affinity Pure Donkey Anti-goat IgG (H + L) (dilution 1:400; #705-165-147; Jackson Immunoresearch, West Grove, PA, USA).

### 2.7. Image Analysis and Statistics

Two independent observers evaluated the specimens blindly. Staining intensities were graded as absent (−), faint (+), moderate (++), or strong (+++). These cut-offs were established by consensus between each investigator following an initial survey of all blindly coded sections. In cases where scoring data differed by more than one unit, the observers re-evaluated the sections to reach a consensus. In other cases, means of the scoring data were calculated.

Observers evaluated between 50 and 200 cells per sample (50 in cartilage, about 100 in Hoffa’s fat and about 200 in synovial samples). All images were captured at the same magnification (40×) and with the same levels of contrast and brightness, scoring them as (−), faint (+), moderate (++), or strong (+++). For statistical analysis, − was quantified as 0, + as 1, ++ as 2, and +++ as 3. Following this, a dependence test (chi-square) was performed between the staining levels of each cell in the three types of tissue studied, and non-parametric techniques (median test and Kruskall-Wallis test) were used to analyze significant differences in the distribution of staining levels with respect to the cell type.

## 3. Results

In order to check the involvement of immunophilin FKBP51, we performed double immunolabelling for FKBP51 and inflammation marker tumor necrosis factor α (TNF-α) and interleukin-6 (IL-6) in tibial plateau cartilage, Hoffa’s fat pad, and synovial tissue sections from patients with advanced knee OA (grade IV). Complementarily, the co-expression levels of this immunophilin along with CD31, Iba1 and CD34 were tested in the same tissues at the protein level. Expression of FKBP51 in sensitive neuronal axons, MAP2+, was also studied in Hoffa’s fat pad.

**Figure 1:**
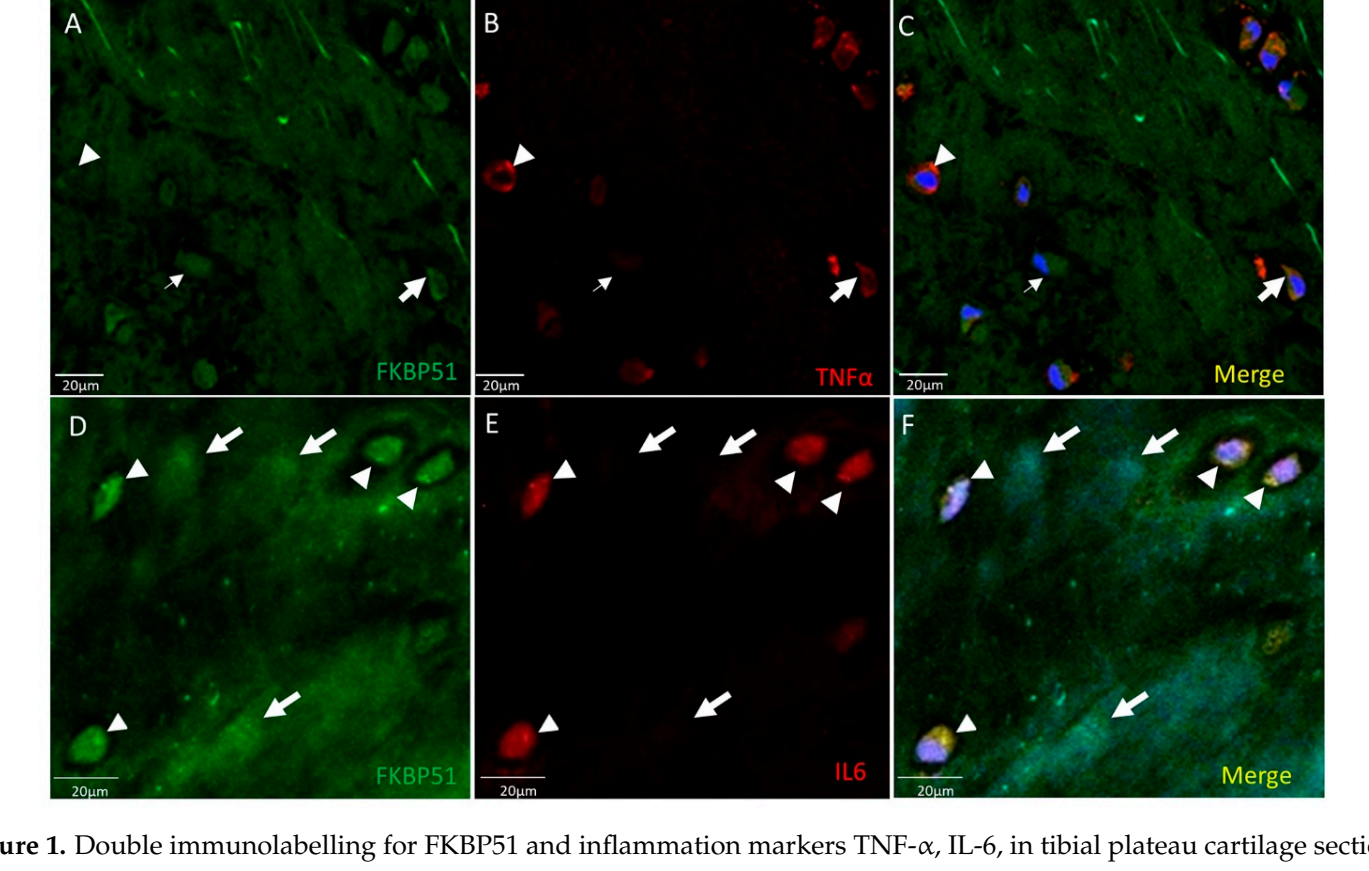
Double immunolabelling for FKBP51 and inflammation markers TNF-α, IL-6, in tibial plateau cartilage sections from a patient with advanced knee OA (grade IV). Merges also contain DAPI labelling. (**A**–**C**) Chondrocytes labeled by FKBP51 antibody at similar intensity levels. Different TNF-α staining intensity was observed in high (arrowhead), medium (bold arrow), and low (thin arrow) positive cells. (**D**–**F**) FKBP51+/IL6+ staining in chondrocytes (arrowheads) and in chondrocytes in background (arrows) within lacunae not cut by microtome.

Positive immunofluorescence for FKBP51 antibody was found in the cytosol of chondrocytes at similar levels, as shown in Figure 1. Different TNF-α staining intensities were observed, high (arrowhead), medium (bold arrow) and low (thin arrow) positive cells, as shown in Figure 1, panel B. Homogeneous staining intensity for FKBP51+/IL-6+ is shown in Figure 1, panels D–F, where arrowheads show FKBP51+/IL-6+ staining in chondrocytes and arrows indicate chondrocytes in the background within lacunae, not cut by microtome.

**Figure 2:**
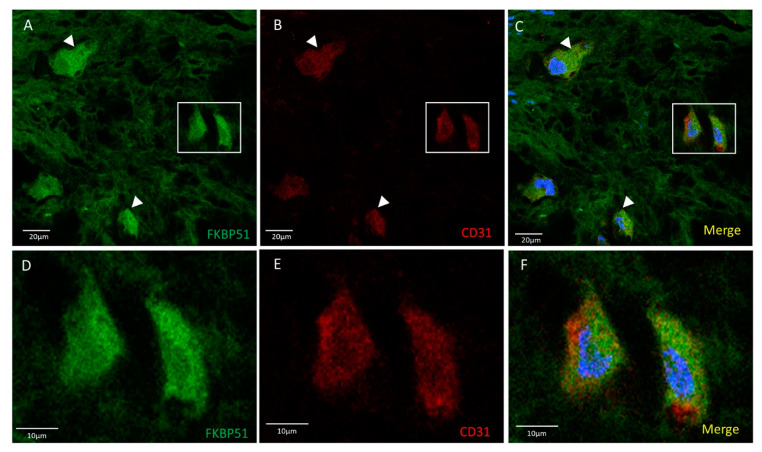
Double immunolabelling for FKBP51 and CD31 in tibial plateau cartilage sections from knee OA grade IV, square for higher magnification of stained chondrocytes (**D**–**F**). Merges also contain DAPI labelling. (**A**–**C**) FKBP51+/CD31+ cells were observed with a homogeneous staining intensity (arrowheads).

Double immunolabelling for FKBP51 and CD31 in tibial plateau cartilage sections from knee OA grade IV showed homogeneous co-staining at medium-high intensity level, more intense in the cytosol of chondrocytes, Figure 2.

**Figure 3:**
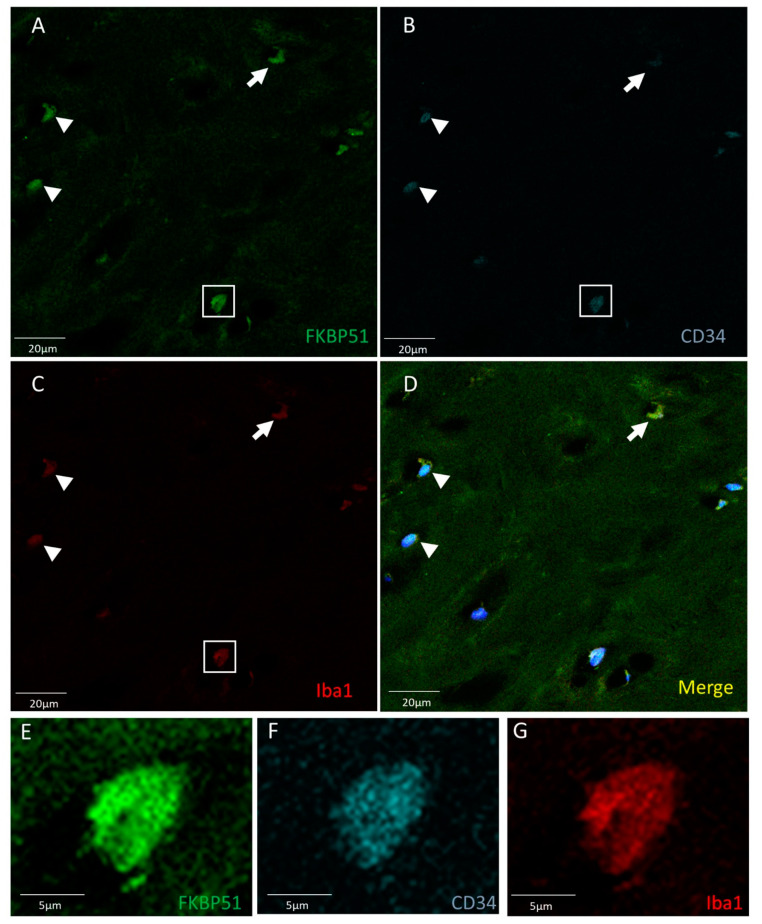
Triple immunolabelling for FKBP51 (green), Iba1 (red), and CD34 (cyan) in tibial plateau cartilage sections from knee OA grade IV, square for higher magnification of a stained chondrocyte (**E**–**G**). FKBP51+/CD34+/Iba+ cells (**A**–**D**) at variable staining intensity regarding CD34 labelling, higher intensity (arrowheads and within the square) and lower intensity (arrow).

Triple immunolabelling for FKBP51, Iba1 (red) and CD34 in tibial plateau cartilage sections from advanced knee OA (grade IV) showed FKBP51+/CD34+/Iba+ immunostained cells, as shown in Figure 3, with more intensity in the cytosol of chondrocytes and with a variable staining intensity regarding CD34 labelling.

**Figure 4:**
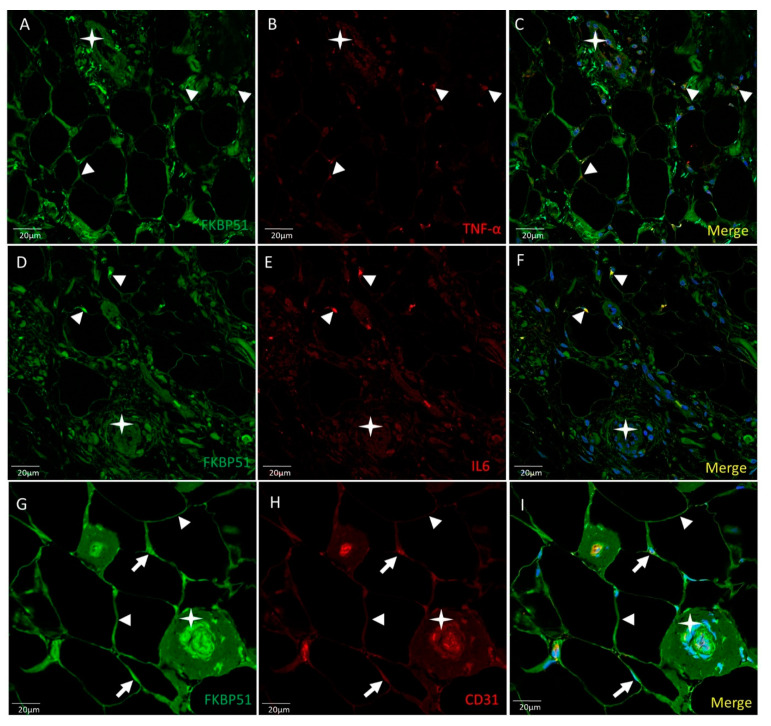
Double immunolabelling for FKBP51 and inflammation markers TNF-α and IL6 and CD31 in Hoffa’s fat pad tissue sections from a patient with knee OA grade IV. Merges also contain DAPI staining. ((**A**–**C**): FKBP51 signal is located at the cytosol and nuclei of all adipocytes. TNF-α staining in adipocytes and tissue around colocalized with FKBP51 (arrowheads). (**D**–**F**): IL6 in adipocytes and tissue around. A small number of cells showed a high staining level colocalized with FKBP51 (arrowheads). Stars are placed in a vessel. (**G**–**I**): FKBP51+/CD31+, immunofluorescence for FKBP51 in cytoplasm of adipocytes (arrowheads) and CD31 mainly around the nuclei (arrows) and in endothelial cells of small vessels.

Double immunolabelling was used for FKBP51 and TNF-α, FKBP51 and IL6, and FKBP51 and CD31 in Hoffa’s fat pad tissue sections from patients with knee OA grade IV. The FKBP51 signal is located in the cytoplasm and nucleus of all adipocytes at a medium or high intensity level. TNFα staining was observed at low level in most adipocytes and surrounding tissue. A small number of cells showed a medium staining level, perinuclear and colocalized with FKBP51 (arrowheads in Figure 4, panels A–C). IL6 (D–F) staining was observed at low level in most adipocytes and surrounding tissue. A small number of cells showed a high staining level, perinuclear and colocalized with FKBP51 (arrowheads). The staining level for small vessels (stars) is very low for both inflammation markers, as shown in Figure 4, panels D–F. FKBP51+/CD31+ (G-H). Immunofluorescence at a medium to high intensity for FKBP51 is located at the cytoplasm (arrowheads) of all adipocytes and CD31 mainly around the nuclei (arrows) and in the endothelium of small vessels, as shown in Figure 4, panels G–I.

**Figure 5:**
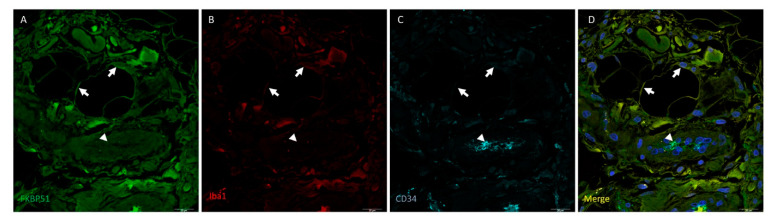
Triple immunolabelling for FKBP51 (green), Iba1 (red) and CD34 (cyan) in Hoffa’s fat pad tissue sections from a patient with knee OA (grade IV). Merges also contain DAPI staining. FKBP51 fluorescence is localized in the cytoplasm and nuclei of virtually all cells at a medium-high intensity level (arrows in **A**). Iba1+ staining in cytosol of adipocytes (arrows). Arrowheads point to FKBP51+/CD34+ endothelial cells ((**A**,**C**,**D**) panels), Iba1- (panel (**B**)).

Triple immunolabelling for FKBP51 (green), Iba1 (red) and CD34 (cyan) in Hoffa’s fat pad tissue sections from patients with knee OA (grade IV) showed FKBP51 fluorescence localized in the cytoplasm and nuclei of many cell types at a medium-high intensity level. Iba1+ staining in cytosol of adipocytes (arrows). Arrowheads point at CD34+ endothelial cells Figure 5, co-stained with FKBP51, but not Iba1 (Figure 5).

**Figure 6:**
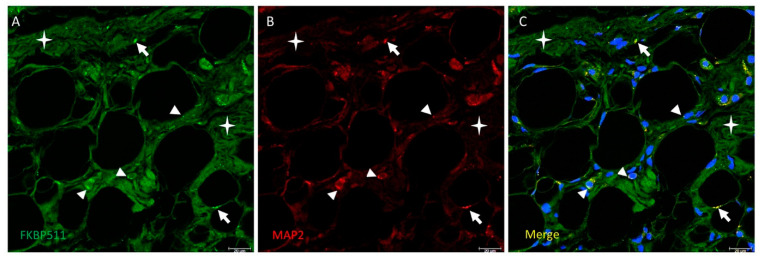
Double immunolabelling for FKBP51 (panel **A**, green) and the microtubule-associated protein 2 (MAP2, panel **B**, red) in Hoffa’s fat pad tissue sections from a patient with advanced knee osteoarthritis (grade IV). Panel **C**: co-staining of FKBP51+/MAP2+ as interspersed dots (arrows), a high number of them passing close to the perinuclear zone of adipocytes (arrowheads). Stars are placed in connective tissue.

In order to check expression of FKBP51 in nerves across Hoffa’s fat pad, double immunolabelling for FKBP51 (green) and MAP2 (red) were performed in tissue sections from patients with advanced knee osteoarthritis (grade IV). Immunofluorescence signal showed co-staining with FKBP51 and MAP2 antibodies, giving an image of interspersed dots predominantly bordering perinuclear areas of adipocytes, Figure 6.

**Figure 7:**
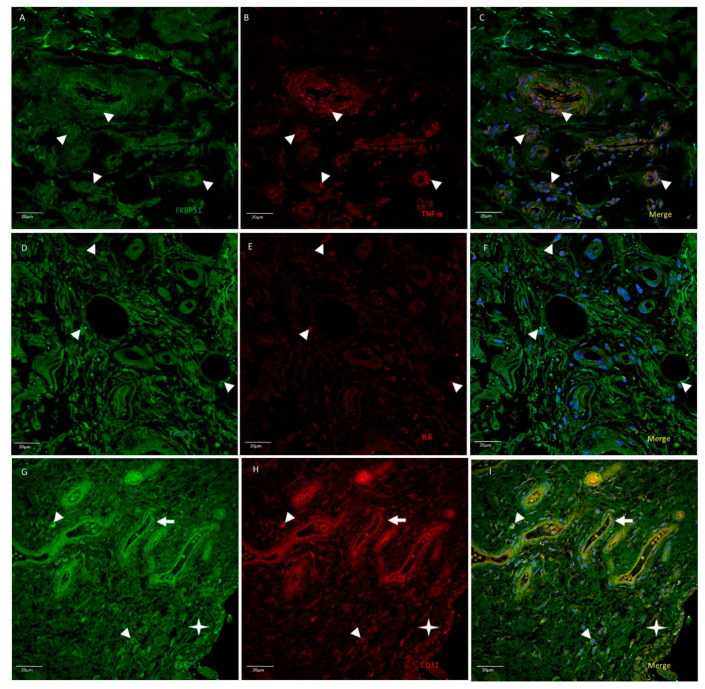
Double immunolabelling for FKBP51 and inflammatory markers TNF-α (panels **A**–**C**) and IL-6 (panels **D**–**F**) and endothelial and white cells marker CD31 (panels **G**–**I**) in synovial tissue sections from a patient with knee OA (grade IV). Merges also contain DAPI staining. Double immunostaining of FKBP51/TNF-α and FKBP51/IL-6 (arrowheads). Panels **G**–**I**: FKBP51+/CD31+ staining in some cells (arrowheads), arrows point to a vessel and the star is placed in the proliferated synovial membrane, showing FKBP51+/CD31+ cells.

In advanced knee osteoarthritis (grade IV), co-immunolabelling for FKBP51 and TNFα at a medium-high intensity, was found in most cells of synovial tissue, including vessels, synovial membrane, and infiltrating cells (Figure 7A–C). Co-staining for FKBP51/IL6 was also found, but to a lesser extent and in only a few cells and locations (Figure 7D–F). FKBP51+/CD31+ fluorescent signal was found in some cells of the synovial tissue, most prominently in the endothelium of vessels and in cells of the proliferated synovial membrane, as shown in Figure 7G–I.

**Figure 8:**
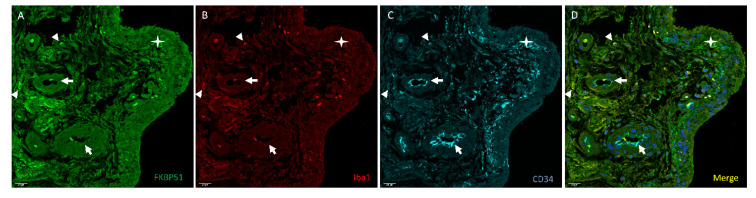
Representative images of triple immunolabelling for FKBP51 (panel **A**), Iba1 (panel **B**) and CD34 (panel **C**) in suprapatellar synovial tissue from a patient with knee OA (grade IV). FKBP51+/Iba+ cytosolic (arrowheads). CD34+ staining was observed in vessels (arrows). The star is placed in the proliferated synovial membrane with several layers of cells. Most cells in the deeper layer are CD34+. Panel **D** shows the merge image of FKBP51, Iba1 and CD34 immunolabelling.

Suprapatellar synovial tissue from patients with advanced knee OA (grade IV) showed few cells with fluorescent signal (red) for the macrophage marker Iba1 (Figure 8, panel B), also positive for FKBP51 and CD34. The mesenchymal-fibroblast progenitor and vascular endothelial marker CD34 (cyan) staining was observed in the endothelium of vessels and in most cells of the inside border of the synovial membrane of the proliferated epithelium (type B synoviocytes). Iba1+ and CD34+ cells are were positive for FKBP51 at different intensities, Figure 8.

Table 2 shows a compilation of the expression levels of FKBP51 and the markers analyzed in different kinds of cells involved in the OA human knee. No significant differences were found among samples from different patients, neither in cellularity nor percentage ratios among the percentages of cells with different expression levels.

## 4. Discussion

Intra-articular corticosteroid injections (IACSI) were first introduced by Dr. J. L. Hollander from the University of Pennsylvania in 1953 for patients with rheumatoid arthritis [42]. Since then, IACSI have become a widely used method for treating pain in patients with knee OA as well, and previous reviews on the topic have generally found it to be an effective treatment, especially in the early and moderate stages [42,43]. However, as the disease progresses, IACSI are shown to be less and less effective and they often produce minimal or non-improvement of symptoms at all in the very advanced stage (grade IV) [41]. The reason for decreasing efficacy is not properly understood [43].

FKBP51 interacts with the glucocorticoid hormone receptor (GR), forming a heterocomplex with Hsp90-Hsp70. Upon binding of the hormone, the steroid receptor heterocomplex exchanges FKBP51 for FKBP52 [44], enhancing the interaction with dynein; then, the complex translocates through nuclear pores to the nucleus in order to activate specific genes [45].

Based on our results and on the findings of previous studies, we can hypothesize that FKBP51 might be involved in the reduction on the IACSI efficacy as the OA disease progresses in the knee joint tissues of these patients. FKBP51 might impair the glucocorticoid signaling process in these patients, leading to a failure in obtaining improvement of symptoms as the disease progresses, maybe by reducing the sensitivity of the GR. Supporting our hypothesis, Kästle et al. showed that FKBP51 silencing in a bronchial epithelial cell line resulted in a 10-fold increase in the effectiveness of dexamethasone therapy related to IL1-beta-induced IL-6 and IL-8, whilst over-expression of FKBP51 significantly reduced the prednisolone sensitivity in a murine pulmonary inflammation model [27]. Samuels J et al. studied the factors related with poor response to IACSI in patients with OA, finding an association between chronic widespread pain and symptoms for depression with a poorer long-term response to IACSI [46]. These results further support our hypothesis that FKBP51 is an important player in the development of knee OA, since the overexpression of this scaffold protein has been previously linked with both chronic pain and depression [33,34,47,48].

The inhibition of the protein FKBP51 in knee OA patients could on the one hand reduce the chronic pain and inflammation, and on the other hand, improve the response to IACSI. This response could be of particular importance in patients with knee OA and depression. In fact, in a search carried out on treatments for depression and post-traumatic stress disorder, it was found that through a screening of 1280 pharmacologically active compounds, three compounds were responsible for attenuating FKBP51-mediated suppression of GR and one disrupted the association of FKBP51 with the GR/Hsp90 [48]. Some of these compounds targeting the FKBP51/GR/Hsp90 complex may be an adjuvant approach in the treatment of this complex pathology.

FKBP51 is a GR negative regulator in adipocytes and its function as a gene expression controller during adipocyte differentiation has been a well-known fact for many years [49,50]. PKA regulates dynamic mitochondrial–nuclear shuttling of FKBP51, which controls the GR target genes involved in the acquisition of adipocyte phenotype [49]. Interestingly, up-regulation of *FKBP5* (the gene encoding FKBP51) expression in human adipose tissue by dexamethasone exposure has been described [51].

In this study we show a frank and consistent expression in all OA samples of CD31, Iba1, and CD34 proteins in the osteoarthritic chondrocytes (Figure 2 and Figure 3, respectively) in a partial co-localization with FKBP51. These findings should be contrasted with previous reports regarding the negative expression of both CD31 and CD34 in chondrogenic progenitor cells derived from human OA knee articular cartilage [52] or in three-dimensional in vitro culture of human chondrocytes derived from osteoarthritis-affected cartilage tissue [53]. The expression of CD31 and CD34 in the synovial membrane of the same OA samples (Figure 7, panel H and Figure 8), reported as negative in synovial membrane mesenchymal stem cells [54], is equally striking. Both CD’s co-localize with FKBP51 (Figure 7).

Our data regarding both chondrocytes and the synovial membrane are in line with those reporting an increased expression of CD31 and CD34 in the knee joints of the Col2α1-Cre; mice by postnatal days 14 and 21 [55]. As both CD31 and CD34 are markers of a number of progenitor cells, their expression in the OA might be an indication of an attempt of the cells to proliferate and differentiate as well as a kind of endochondral bone formation [55].

In our study we found a significant amount of the FKBP51 protein expression in the joint tissues of advanced osteoarthritic knees. In this context, it is important to consider that all the samples were taken from a very homogeneous group of patients presenting advanced stage knee OA. All patients presented severe chronic pain and disability. This is of particular interest as the protein FKBP51 has previously been linked to chronic pain and inflammation [32]. However, a direct link between pain and FKBP51 expression cannot be proposed from the data and results of the present study. We can only hypothesize about this potential relationship. The expression of FKBP51 in Hoffa’s fat pad nerves (Figure 6) may show a point for therapy, furthermore, considering that some studies show that when Hoffa’s fat pad was completely removed during total knee arthroplasty for osteoarthritis or rheumatoid arthritis, those patients presented with a higher rate of persistent pain one year after surgery [56]

As reported by Maiarù M. et al. [34], FKBP51 is a major culprit in the development and maintenance of long-term pain states and, furthermore, in FKBP51 knockout mice and in those with the FKBP51 silenced gene in the spinal cord, showed reduced sensitivity in several persistent pain models in those rodents. Indeed, FKBP51 deletion did not compromise the detection of acute painful stimuli. Moreover, the intrathecal administration of the specific FKBP51 inhibitor SAFit2 reduced the severity of an established pain state, confirming the crucial role of spinal FKBP51 in nociceptive processing [32,33]. In addition, glucocorticoid signaling, which is known to modulate persistent pain states in rodents, was impaired in FKBP51 knockout mice. This finding suggested that FKBP51 regulates chronic pain through the modulation of glucocorticoid signaling, especially considering the crucial role of FKBP51 in the regulation of the GR activity through its interaction as a co-chaperon of Hsp90 [32]. However, no links have been established between FKBP51 and chronic pain in OA and further studies are still needed in order to overcome the limitations mentioned and to gain a deeper understanding of the role of FKBP51 in the pathophysiology of knee OA.

## 5. Conclusions

In conclusion, our results clearly demonstrate co-expression of FKBP51 with TNF-α, IL-6, CD31 and CD34 in chondrocytes, synovial membrane cells and adipocytes in Hoffa’s fat pad from OA joints. In addition to this, FKBP51 was abundantly expressed in nerve fibers within the fat pad. Co-expression of FKBP51 protein with these markers may be indicative of its contribution to inflammatory processes and might be involved in the transition from inflammation to degeneration in OA. However, further studies need to be done in order to establish variation in this scaffold protein expression, starting with a young healthy knee and ending with an end of stage one, including all the different stages of OA.

## 6. Limitations

This study highlights the cellular and subcellular expression levels of FKBP51 and its possible relationship with inflammatory markers in the human osteoarthritic knee at a final stage. Consequently, this study is limited by the lack of data regarding healthy controls and those in previous stages or without either inflammation or degeneration, for which surgical procedures are not indicated. Experimental animal models could shine some light on those stages, but neither mechanical nor biological circumstances are similar to that of the real human OA disease.

## Figures and Tables

**Table 1:** Clinical and demographic data of the patients enrolled. Kellgren & Lawrence classification for osteoarthritis (K&L OA Grade) [41].

Number	Gender	Age	BMI	K&L OA Grade
1	F	66	32	IV
2	M	81	32.5	IV
3	F	52	24.6	IV
4	F	56	34.2	IV
5	M	70	35.3	IV
6	F	72	31.1	IV
7	M	59	31.6	IV
8	F	59	43.1	IV
9	F	76	29.4	IV
10	F	69	30.8	IV
11	F	72	30.4	IV
12	F	69	27.1	IV
13	F	52	33	IV
14	M	75	33.5	IV

**Table 2:** Levels of specific fluorescence signal for FKBP51, TNF-α, IL-6, Iba1, CD31, CD34, and MAP2 in different cell types of tissues from patients with advanced knee OA (grade IV). Intensity levels: − no expression, + faint, ++ medium and +++ high. nc, not checked.

	FKBP51	TNF-α	IL6	Iba1	CD31	CD34	MAP2
Chondrocytes	++/+++	+/+++	+++	++	++/+++	+/++	nc
Adipocytes	++/+++	+/++	+/+++	+/++	+	+	+
Synoviocytes (A type)	++/+++	++	+	+	+	+	nc
Synoviocytes (B type)	++/+++	++	+	++	++	+++	nc
Axon fibers	++	nc	nc	nc	nc	nc	+++
Endothelial cells	++/+++	++/+++	+	−	+++	+++	nc
Vascular endothelial cells	++/+++	+/+++	+	−	+/++	++/+++	nc
